# Use of Cyclosporine and Itraconazole as Palliative Treatment for Proventricular Dilatation Disease in Psittacine Birds

**DOI:** 10.3390/vetsci12050459

**Published:** 2025-05-12

**Authors:** Laura M. Kleinschmidt, Sharman M. Hoppes, Jeffrey M. B. Musser, Ian Tizard, J. Jill Heatley

**Affiliations:** 1 Saint Louis Zoo, One Government Drive, Saint Louis, MO 63110, USA; 2 Department of Small Animal Veterinary Clinical Sciences, Texas A&M University, College Station, TX 77845, USA; 3 Department of Veterinary Pathobiology, MS #4456, Texas A&M University, College Station, TX 77843, USA

**Keywords:** immunosuppressant, Proventricular Dilatation Disease (PDD), Psittacine Bornavirus (PaBV), Psittaciformes, avian, bornavirus, parrot

## Abstract

Proventricular dilatation disease (PDD) is a neurologic syndrome of birds caused by the infectious agent Psittacine Bornavirus (PaBV). Clinical disease may be based on the T-cell-mediated immune response to PaBV within the central and peripheral nervous system, similar to Borna disease virus, a closely related mammalian virus. Lymphoplasmacytic infiltrations may occur in ganglia, nerve plexuses, peripheral nerves and the central nervous system of the infected bird. Clinical disease may result in multiple neurologic disorders and life-threatening morbidity. Treatment of PDD with antivirals and non-steroidal anti-inflammatories has thus far been non-curative and unsuccessful long-term. Cyclosporine is an immunosuppressant drug that decreases cell-mediated immune responses by inhibiting T-cell proliferation and decreasing cytokine production. In avian species, cyclosporine is a potent immunosuppressant with T-cell-specific action. A pilot study performed in PaBV-infected cockatiels showed increased weight gain and a lack of morbidity or mortality following experimental PaBV infection and cyclosporine treatment at 10 mg/kg orally every 12 h. In this case series of six psittacine birds affected by PDD, cyclosporine at this dose alleviated or reduced clinical signs in multiple birds without severe sequelae. Itraconazole was used concurrently in these cases to prevent secondary fungal infections during immunosuppression but may have had a synergetic effect when used in combination with cyclosporine. Further prospective research is indicated to better evaluate cyclosporine use in birds with PDD. However, these preliminary clinical findings suggest that cyclosporine and itraconazole administration is a treatment option for palliation of PDD in psittacine patients, especially those refractory to other treatments.

## Simple Summary

Proventricular dilatation disease (PDD) is a neurologic syndrome of birds caused by the infectious agent Psittacine Bornavirus (PaBV). Clinical disease may be based on T-cell-mediated immune response to PaBV within the central and peripheral nervous system. Clinical disease may result in multiple neurologic disorders and life-threatening morbidity. Treatment of PDD with antivirals and non-steroidal anti-inflammatories has thus far been non-curative and unsuccessful long-term. Cyclosporine is an immunosuppressant drug that decreases cell-mediated immune responses by inhibiting T-cell proliferation and decreasing cytokine production. In avian species, cyclosporine is a potent immunosuppressant with T-cell-specific action. A pilot study performed in PaBV-infected cockatiels showed increased weight gain and a lack of morbidity or mortality following experimental PaBV infection and cyclosporine treatment at 10 mg/kg orally every 12 h. In this case series of six psittacine birds affected by PDD, cyclosporine at this dose alleviated or reduced clinical signs in multiple birds without severe sequelae. Further prospective research is indicated to better evaluate cyclosporine use in birds with PDD.

## 1. Introduction

This clinical case series evaluated cyclosporine as a palliative treatment for birds affected by proventricular dilatation disease (PDD). PDD is a neurologic syndrome of birds caused by the infectious agent Psittacine Bornavirus (PaBV) [1,2,3,4,5,6]. PaBV is a neurotropic virus with a predilection for gastrointestinal ganglia, but it can affect many nerves throughout the body [7]. Over 80 species of psittaciformes as well as passeriformes, coliiformes, pelecaniformes, piciformes, anseriformes, falconiformes, and accipitriformes have been reported to be infected with avian bornaviruses [2,3,6,8,9,10,11,12,13,14,15]. Proposed transmission routes include vertical transmission (eggs) and horizontal transmission of viral particles in respiratory secretions, feather dust, feces and urine [2,3,6,16,17]. The presence of PaBV does not always result in clinical disease; the time between infection and presentation of clinical disease is variable, and some avian species appear to clear of or tolerate the virus without clinical disease [2-3Clinical disease is thought to be based on the T-cell-mediated immune response to the presence of PaBV within the nervous system, similar to Borna disease virus (BDV), a closely related mammalian virus with a similar immune-mediated mechanism of disease production [2,3,6,14,18,19,20]. Lymphoplasmacytic infiltrates develop in affected enteric ganglia, enteric nerve plexuses, brachial, vagus, optic, and sciatic nerves and/or the central nervous system [2,11,13,14,18,19,20,21]. Clinical disease presentations include gastrointestinal and neurologic clinical signs [2,6,11,13]. Gastrointestinal clinical signs include regurgitation, delayed crop emptying, poor gastrointestinal motility, enteric bacterial overgrowth or dysbiosis, diarrhea, voluminous or gas-containing stools, and, rarely, the passage of undigested seeds or other foodstuffs [2,6,19]. Neurologic clinical signs include behavioral changes such as seizures or tremors, blindness, and ataxia manifested as gait change or inability to grip or perch normally [3,6,19,22]. Definitive diagnosis of PDD may be challenging antemortem. Classic diagnostic findings include a dilated proventriculus and/or other portions of the gastrointestinal tract on lateral radiograph, PaBV-positive RT-PCR (reverse transcriptase polymerase chain reaction) testing of cloacal swabs, positive serological reaction, and/or histopathologic lymphoplasmacytic infiltrates within the myenteric ganglia and nerves of the central, peripheral, or autonomic nervous system on necropsy [2,6,11,13,14,18,19,20,23]. Treatment of PDD has thus far been non-curative and unsuccessful long-term, although treatment modalities are still being actively investigated [2,3,20,24]. Antiviral therapies have not been demonstrated to be effective in the treatment of PDD [2,3,20]. Non-steroidal anti-inflammatories (NSAIDs) including meloxicam and celecoxib have been used historically with variable results [2,3,24]. To limit the progression of disease and decrease the morbidity and mortality associated with PDD in avian patients, new treatment options must be explored.

Cyclosporine decreases cell-mediated immune responses by inhibiting T-cell proliferation via calcineurin inhibition of growth cycles and decreasing cytokine production [3,25]. Cyclosporine has been used to treat immune-mediated and inflammatory diseases in veterinary medicine, including inflammatory bowel disease, atopic dermatitis, perianal fistulas, hemolytic anemia, thrombocytopenia, and uveitis [25]. Known side effects of cyclosporine include leukopenia secondary to immunosuppression, hepatotoxicity, and nephrotoxicity [25]. Cyclosporine has been used in experimental treatment of BDV in rats with promising results [7,26,27,28]. Cyclosporine is a proven potent immunosuppressant with T-cell-specific action in avian species that has been used to induce immunosuppression at doses up to 100 mg/kg intramuscularly in gallinaceous birds [29,30,31,32,33,34,35,36,37,38,39,40,41,42,43,44]. Bioavailability of cyclosporine varies based on the route of administration and formulation; thus, compounded formulations of cyclosporine cannot be recommended based on possible bioavailability differences [25]. In Pekin ducks, a pharmacokinetic study revealed that cyclosporine was metabolized and cleared faster than in a comparative mammalian species; therapeutic plasma levels in the bloodstream were maintained for less than 8 h following 10 mg/kg intravenous dosing [45]. Thus, increasing the dose to at least 60 mg/kg every 24 h or decreasing the dose interval to every 6–8 h was recommended for cyclosporine use in avian species [45]. Unfortunately, a high dose, 3–4 times daily dosing [45], is not reasonable for most parrot owners to accomplish and could interfere with the human–parrot bond. Additional pharmacokinetic or pharmacodynamics studies to assess the best dosing protocols have not been performed in avian species.

Immunosuppressant effects of cyclosporine could increase the likelihood of fungal infections such as aspergillosis; thus, in the clinical case series presented, low-dose itraconazole was given prophylactically. Itraconazole increases plasma concentrations of cyclosporine via the induction of liver enzymes (cytochrome P450) in animals, as occurs for other azole drugs in vitro [25,36]. Azole antifungals have been used in dogs to reduce the necessary dose of cyclosporine [25]. Therefore, itraconazole may have a synergistic effect when used in combination with cyclosporine [15,46,47]. Itraconazole was chosen based on commercially available liquid formulations, but other antifungal azole drugs may also be acceptable for this purpose as well as aspergillosis prophylaxis. However, the synergism of any azole drugs with cyclosporine is ultimately unproven in birds.

The purpose of this clinical case series was to evaluate cyclosporine treatment as a palliative treatment for birds affected with PDD, which has been pursued at the Texas A&M University Zoological Medicine Service for more than twenty years. In large part, the dosing protocols shared here were based on a pilot study in experimentally PaBV-infected cockatiels; healthy adult cockatiels had been inoculated by intramuscular and oral routes with the M24 strain of PaBV genotype 4, previously shown to induce PDD in cockatiels [22,24]. These studies were reviewed by the Texas A&M University Institutional Animal Care and Use Committee and conducted based on approved animal use protocol #2011-024. Control, meloxicam, and cyclosporine treatment groups of four parrots each were either not treated, dosed with 0.5 mg/kg meloxicam orally every 12 h, or dosed with cyclosporine at 10 mg/kg orally every 12 h, respectively. Birds treated with meloxicam became ill within the first month, showing clinical signs of weight loss, regurgitation, and lethargy, and died or were euthanized based on the severity of symptoms on days 61, 78, 95 and 117 [24]. Necropsy findings were consistent with PDD including poor body condition and an enlarged proventriculus and ventriculus [24]. Mild encephalitis and ganglioneuritis, comprised of lymphoplasmacytic infiltrates, occurred within the ventriculus and heart; most of the 15–19 tissues tested were RT-PCR-positive for PaBV in the four birds. Based on these unexpected results in the meloxicam-treated PaBV-infected birds, a separate control study in four healthy cockatiels was performed with the same dose of meloxicam but without inoculation with PaBV [24]. These birds lacked signs of illness throughout the study and, on necropsy, had occasional lymphoid foci in the liver but lacked lesions suggestive of PDD. One bird was PCR-positive for PaBV only within the adrenal gland. In the experimentally PaBV-infected cockatiels, control and cyclosporine-treated birds remained clinically healthy during the study. No untoward clinical signs were noted during cyclosporine treatment, and treated and control birds were euthanized at 153 and 151 days for necropsy, respectively. Compared to the control group, birds that received cyclosporine had increased in weight by the end of the study. Three cyclosporine-treated birds had subtle histologic lesions of PDD on necropsy, and all 19 tissues tested were RT-PCR-positive for PaBV in all birds. Of the four control birds, one bird had histologic lesions consistent with PDD, and in two birds, 19 of 19 tissues sampled were PCR-positive for PaBV, while the remaining two birds had 6 out of 19 tissues PCR-positive for PaBV. In this pilot study, neither meloxicam nor cyclosporine decreased the organ PCR-positive rate for cockatiels infected with PaBV, but cyclosporine was effective in preventing the manifestation of PDD clinical signs [22,24]. Based on these preliminary studies, cyclosporine and itraconazole treatment was used as a palliative option in birds with severe clinical signs of PDD that had not responded to treatment with NSAIDs. We report six of these cases below, which were exempted from ethical review based on Texas A&M University College of Veterinary Medicine guidelines and based on alignment with ARRIVE 2.0 guidelines for transparent reporting of animal case studies.

## 2. Case Series

This report describes clinical cases in which cyclosporine was used to treat proventricular dilatation disease (PDD)—associated clinical signs in multiple large psittacine birds at Texas A&M University Zoological Medicine Service (TAMU-ZMS). The cyclosporine used in these cases was the commercially available liquid formulation Atopica^®^ (Elanco Animal Health, IN, USA). Signalment, clinical presentation, flock history, diagnostics, treatment, and outcomes are presented below. Cloacal swab testing via PaBV RT-PCR and serologic testing was performed at the Schubot Exotic Bird Health Center Laboratory (Table 1); all RT-PCR-positive cases were genotype PaBV-4. Parameters monitored for health assessment before and during treatment in each case are provided in Table 1 and Table 2. For all cases, a complete blood count (CBC) and plasma biochemistry panel were recommended before beginning treatment, and at two weeks, 1, 2, and 3 months after beginning treatment to monitor white blood cell counts and organ function. Medical progress checks to include CBC and plasma biochemistries were then recommended quarterly during the course of treatment. However, financial concerns may have limited client compliance with these recommendations.

### 2.1. Case 1: Green Wing Macaw (Ara Chloropterus)

A juvenile intact male green wing macaw was donated to Schubot Exotic Bird Health Center in 2007 with a history of exposure to birds affected by PDD. At the time of donation, the individual showed no clinical signs of PDD, was RT-PCR- and Western-blot-negative, and was otherwise apparently healthy. Crop biopsy findings were consistent with a diagnosis of PDD. In 2011, the macaw became acutely ill. During a routine exam three weeks prior to the onset of clinical illness, the patient had been clinically normal and serologically negative for PaBV. The patient was hospitalized, and supportive care was initiated. Injectable meloxicam (0.5 mg/kg BID, Metacam, Boehringer Ingelheim, St. Joseph, MO 64506, USA) was given for two days without any clinical effect. On day three, cyclosporine (10 mg/kg PO BID, Atopica, Elanco, Greenfield, IN 46140, USA) and celecoxib (10 mg/kg PO SID, Celebrex, Pfizer, New York, NY 10017, USA, compounded by Texas A&M University Small Animal Hospital Pharmacy, College Station, TX 77845, USA) were initiated. These treatments were continued for three days, but the patient continued to decline despite treatment. The patient was ultimately euthanized and submitted for necropsy. Necropsy results revealed histologic evidence of PDD in multiple organs including the gastrointestinal tract. Serology testing and RT-PCR of samples taken at necropsy were positive for PaBV.

### 2.2. Case 2: Umbrella Cockatoo (Cacatua Alba)

A 4-year-old intact female umbrella cockatoo presented to TAMU-ZMS in June 2013 with the complaint of increased chick-like behavior progressing to regurgitation and weight loss. The patient had been exposed to two other birds in the household that had died from PDD in March and May of 2013. A cloacal/choanal swab was RT-PCR-positive for PaBV. The patient was started on cyclosporine (10 mg/kg PO BID), itraconazole (2.5 mg/kg PO SID, Sporanox, Titusville, NJ 08560, USA), and meloxicam (0.5 mg/kg PO BID, Metacam, Boehringer Ingelheim, St. Joseph, MO 64506, USA). The owner gave the medications for approximately 1 month but felt that the medications had not made a clinical difference. However, clinical examination after the treatment period revealed no change in body condition score and an increase in body weight compared to the start of treatment. Based on concerns about quality of life, the owner elected for humane euthanasia. On necropsy, histologic lesions consistent with PDD were present in the gastrointestinal tract, but no lesions were noted in other systems. The brain was PaBV RT-PCR-positive.

### 2.3. Case 3: Umbrella Cockatoo (Cacatua Alba)

A 4-year-old intact female umbrella cockatoo was donated to the Schubot Exotic Bird Health Center in 2007 based on exposure to PaBV. The patient was clinically ill on arrival, with a low body condition score and occasional regurgitation. Histological examination of crop biopsy revealed histologic lesions consistent with PDD. RT-PCR and serology were also positive for PaBV at the time of presentation. The patient was started on meloxicam (0.5 mg/kg PO BID). The individual was also hand-fed to maintain weight once to twice daily as needed for four years. In 2009, the patient developed a dilated proventriculus on radiographs. In June 2010, the patient presented to TAMU thin, regurgitating, depressed, dehydrated, and losing weight. Complete blood count revealed leukocytosis, and a review of radiographic images revealed persistent proventricular dilatation. The administration of cyclosporine (5 mg/kg PO BID), itraconazole (2.5 mg/kg PO SID), and marbofloxacin (5 mg/kg PO SID, Zeniquin, Zoetis, Parsippany, NJ 07054, USA) was initiated. The patient improved over the next three weeks, with returned appetite, discontinued regurgitation, and weight gain. A recheck of the complete blood count revealed a decrease in leukocytosis, and a review of radiographic images showed decreased proventricular dilation. Five weeks following the start of cyclosporine, the patient presented again to TAMU weak, dehydrated, and regurgitating. Supportive care was initiated, but the patient died two days following presentation. Necropsy revealed PDD lesions through the GI tract and brain. PaBV RT-PCR of multiple organs at the time of necropsy was positive for PaBV. Although the patient had an active case of PDD, gout also severely affected the heart and pericardial sac and was widely disseminated. A combination of chronic administration of non-steroidal anti-inflammatories, long-term hand-feeding formulas, and the initiation of cyclosporine may have contributed to renal damage and, thus, the development of gout and ultimately death in this patient.

### 2.4. Case 4: Green Wing Macaw (Ara Chloropterus)

A juvenile intact male green wing macaw, a sibling of case 1, was donated to Schubot Exotic Bird Health Center in 2007 with a history of exposure to birds affected by PDD. At the time of donation, the individual was RT-PCR- and serology-positive for PaBV; histologic findings from crop biopsy were consistent with PDD. In 2011, caretakers noted that the patient was not performing his normal training behaviors. Radiographs revealed mild enlargement of the proventriculus. Despite the administration of meloxicam (0.5 mg/kg PO BID) for two weeks, improvement was not evident. Therefore, cyclosporine (10 mg/kg PO BID) and itraconazole (2.5 mg/kg PO SID) were initiated. The patient showed improvement to its normal attitude within 3 days of the initiation of treatment, gained weight, and the proventricular size was reduced on a recheck of the radiographs. After 3 months, the medications were discontinued. The patient continues to thrive at the Schubot Exotic Bird Health Center at the time of this publication.

### 2.5. Case 5: Red-Fronted Macaw (Ara Rubrogenys)

An 8-year-old intact male red-fronted macaw presented to his referring veterinarian in 2012 with a history of being PaBV RT-PCR-positive for several years and a history of multiple PDD associated deaths within his flock over several years. He was started on celecoxib (10 mg/kg PO SID) and was well for one year. However, he presented to TAMU in 2013 when he became depressed, began regurgitating occasionally, and had a decreased appetite. Radiographs revealed a dilated proventriculus. Plasma biochemistries and CBC failed to reveal abnormalities. The body condition score was graded a 1+ out of 5. Cyclosporine (10 mg/kg PO BID) and itraconazole (2.5 mg/kg PO SID) were initiated. The owner reported that within one week of starting treatment, the patient was eating better, gaining weight, and returned to its normal attitude and behaviors. After 3 months of treatment, both medications were prescribed to be administered once daily. Since that time, the patient has been on cyclosporine at a 10 mg/kg daily dose prophylactically. The owners have declined discontinuation of the medications. The patient continues to thrive at home at the time of this publication.

### 2.6. Case 6: Blue and Gold Macaw (Ara Ararauna)

A 5-year-old intact male blue and gold macaw presented to TAMU in January 2015 with a three day history of regurgitation and lethargy. This individual was part of a flock affected by PaBV, with two PDD-associated deaths in macaw cage-mates and a PaBV positive sun conure currently housed in the same room. Radiographs revealed an enlarged proventriculus. Plasma biochemistry and complete blood count abnormalities were limited to increased concentrations of creatine kinase. We attributed these increased concentrations to handling and intramuscular midazolam injection prior to sample collection. Testing for ABV via RT-PCR was negative at this time, likely based on intermittent viral shedding. The owner declined any further diagnostics. Based on the history of PDD-associated flock deaths and likelihood of PaBV exposure, the owner elected to move forward with treatment for suspected PDD. The macaw was started on meloxicam (0.5 mg/kg PO BID), enrofloxacin (21.4 mg/kg PO SID, Baytril, 50 mg/mL oral suspension, compounded by Texas A&M University Small Animal Hospital Pharmacy, College Station, TX 77845, USA), itraconazole (2.5 mg/kg PO SID), and cyclosporine (10 mg/kg PO BID). The owner noted the resolution of regurgitation and an improved attitude within 2 weeks of treatment initiation. Cycloporine dosing was reduced to once daily for 1–2 months following the initiation of treatment. The recheck examination and complete blood cell count approximately 3 months after the initiation of treatment revealed mild leukopenia as the only significant abnormality. Therefore, the cyclosporine dosing interval was increased to every 48 h. The patient continues to thrive with no recurrence of symptoms at the time of writing this paper.

## 3. Discussion

This case series suggests cyclosporine as a viable palliative treatment option for PDD in psittacine birds. In six clinical cases of psittacine birds affected by PDD, the use of cyclosporine reduced clinical signs and may have prevented the progression of PDD in multiple birds. Proventricular dilatation disease was diagnosed antemortem in these cases via a combination of RT-PCR PaBV-positive cloacal swabs, consistent clinical signs, lack of evidence of other etiologies of clinical signs, positive serological response, history of exposure to PaBV, and/or crop biopsy. PDD diagnosis was confirmed on necropsy for cases 1–3. Although histopathologic lesions confirm a diagnosis of PDD, antemortem crop biopsies may not be positive in all PDD cases, as lymphoplasmacytic infiltrate more commonly affects the proventriculus and ventriculus [2,6,11,13,14,18,19,20]. Thus, a clinical or presumptive diagnosis may be reached using the aforementioned criteria, as well as standard screening tests of CBC, biochemistry panel and fecal cytology and culture, in order to treat affected birds prior to death. Psittacine bornavirus is intermittently shed via the gastrointestinal tract and can thus be detected via RT-PCR testing. A PDD-affected bird may be negative on a single or multiple RT-PCR screening test as viral shedding is not known to be predictive for disease (PDD). In addition, based on our clinical experience, even clinical disease does not guarantee viral shedding of PaBV. Repeated PCR tests and serological testing are recommended if a bird is suspected of having PDD but is negative on initial PCR.

Antifungals were used to prevent secondary fungal infections based on the concern of immunosuppression in these cases, though the synergetic effect when used in combination with cyclosporine cannot be ruled out [25]. Long-term usage of itraconazole should be considered judiciously to discourage the development of itraconazole resistance. Cyclosporine at 10 mg/kg orally every 12 h was administered long-term in several cases without significant side effects. Side effects directly attributable to cyclosporine treatment appear to be limited to mild leukopenia in one case. Non-steroidal anti-inflammatory drugs (NSAIDs) were used in conjunction with cyclosporine and itraconazole in some cases to reduce secondary inflammatory prostaglandins rather than treat the primary immune-mediated disease process directly. Despite favorable pharmacokinetic data for some, we continue to find only anecdotal support in the literature to support NSAIDs for the treatment of PDD [48]. Controlled studies of PaBV infections have continued to fail to find support for the therapeutic use of NSAIDs for the mitigation of PDD or ABV infection [49,50]. Three cases (cases 3–5) that became refractory to treatment with an NSAID alone responded positively to cyclosporine and itraconazole treatment. Antibiotics were used on a case-by-case basis at clinician discretion and were generally based on flora overgrowth of the GI tract as diagnosed by culture or fecal cytology.

Three parrots (cases 4–6) underwent treatment and continue to thrive at the time of writing this paper. Of the three parrots that are no longer living, only one was completely non-responsive to treatment (case 1). Because this patient only received cyclosporine for 3 days, therapeutic or toxic levels were unlikely to have been achieved. The second case improved clinically based on weight gain after one month of treatment. However, because the owner elected for euthanasia, long-term effects of cyclosporine treatment in that patient are unknown. The third case initially responded to treatment, with normalized clinical status prior to decline five weeks after the initiation of treatment. Interestingly, the histopathologic exam of tissues at necropsy indicated gout as the cause of death in this patient, rather than PDD. The authors postulate that gout occurred in this patient due to long-term use of high-protein hand feeding formulas (intended for short-term use in the young of debilitated birds), chronic use of NSAIDs (which can cause renal toxicity in birds at high doses) [51], and the addition of cyclosporine, which is a nephrotoxin in mammals [25,52].

Psittacine birds affected by PDD and their owners and caretakers are faced with the possibility of what should be a long-lived companion species suffering a slow downward spiral of a wasting and neurologic disease ending in death. Despite excellent medical and husbandry management, parrots suffering from PDD may have refractory clinical illness. We suggest that, while better prevention and treatment continue to be researched, cyclosporine is a valid palliative option for management of this disease in severely clinically affected parrots. However, close clinical monitoring must be maintained during treatment due to the risks of side effects (leukopenia, hepatotoxicity, nephrotoxicity), which may increase in likelihood with chronic use of these drugs. Limitations of this study include the limited sample size, variations in individual case management, and lack of available pharmacokinetic studies of cyclosporine in Psittaciformes. Based on limited trials, increased PCR positivity and shedding of PaBV may occur after cyclosporine treatment. Additional research is recommended to further evaluate the use of cyclosporine in birds with PDD. Pharmacodynamic, pharmacokinetic, and toxicity studies would be useful to further evaluate therapeutic levels and dosing intervals of cyclosporine in parrots.

## Figures and Tables

**Table 1:** Health parameters of psittacine patients undergoing cyclosporine and itraconazole treatment.

Case	Survival Time	Cyclosporine Duration	Body Condition Score		Patient Body Weight (g)		Proventriculus: Keel Ratio		PaBV RT-PCR		Gross Necropsy	Histopathological Lesions of PDD		
Number	Days (d)		Pre	Post	Pre	Post	Pre	Post	Pre	Post	Lesions	Brain	Gastrointestinal	Kidney
1	3 d	3 d	3	2	1080	ND	ND	ND	−	+	PDD	ND	Yes	ND
2	42 d	42 d	2	2.5	402	440	0.72	ND	+	+	PDD	No	Yes	No
3	82 d	82 d	2	1.5	490	432	0.72	0.5	+	+	Gout, PDD	Yes	Yes	ND
4	1825 d	92 d	2	4	1320	1660	0.7	0.5	+	+	NA	NA	NA	NA
5	913 d	913 d	1+	4	422	482	0.64	0.52	+	+	NA	NA	NA	NA
6	400 d	400 d	3.5	4	1170	1180	0.84	ND	−	−	NA	NA	NA	NA

Abbreviations: ND, not determined; NA, not applicable; +, Positive; −, Negative; Pre, values determined prior to cyclosporine treatment; Post, values determined after cyclosporine treatment; PDD, lesions of proventricular dilatation disease (PDD) were present; Gout, lesions of gout were present; Yes, histopathological lesions of PDD were present; No, no histopathological lesions of PDD were present. Survival Time, length of survival in days post-initiation of cyclosporine treatment at time of writing this paper.

**Table 2:** Complete blood count and plasma biochemical parameters before and after cyclosporine treatment in parrots affected by proventricular dilatation disease. Cases 5–6 include the most recent post-initiation of treatment hematological and biochemical values.

Case Number	Sample Time	WBC Total	Hetero	Lymph	Mono	Eos	Baso	PCV	AST	BA	UA	GLU	TP	ALB	GLOB
		Cells × 10^3^/µL			Cells/µL			%	U/L	µmol/L	mg/dL	mg/dL	g/dL	g/dL	g/dL
1	0	37.1	25.228	10.759	371	371	371	45	152	<35	8.6	272	3.9	2.7	1.1
2	0	26.7	3.204	21.627	1602	267	267	34	135	53	3.5	278	2.8	1.8	1.0
	42 d	17.8	10.502	7.12	178	0	0	28	98	<35	5.0	281	2.1	1.4	0.7
3	0	26.7	20.025	4.005	1869	267	534	40	132	<35	18.3	216	2.5	1.4	1.2
	47 d	17.8	13.706	3.560	534	0	0	40	106	<35	9.9	250	2.4	1.4	1.0
4	0	30.5	21.655	7.015	1220	0	610	40	205	<35	4.4	291	3.6	3.2	0.4
	1890 d	20.2	15.958	3.232	808	202	0	45	426	<35	3.2	310	4.1	3.3	0.8
5	0	24.5	17.885	4.410	1960	245	245	45	171	<35	7.7	303	4.7	2.4	2.3
	560 d	12.9	10.965	1.419	387	0	129	45	140	<35	7.3	295	3.2	2.4	0.9
6	0	16.3	10.432	4.075	1630	0	163	49	86	<35	2.5	275	3.6	2.5	1.2
	94 d	6.7	4.221	2.144	201	0	134	48	173	<35	4.1	282	4.4	3.3	1.1

Abbreviations: WBC, leukocyte; Hetero, heterophil; Lymph, lymphocyte; Mono, monocyte; Eos, eosinophil; Baso, basophil; PCV, packed cell volume; AST, aspartate aminotransferase; BA, bile acids; UA, uric acid; GLU, glucose; TP, total protein; ALB, albumin; GLOB, globulin; Sample time, evaluated prior to (T = 0) and after cyclosporine treatment (days (d)) following initiation of treatment.

## Data Availability

The raw data supporting the conclusions of this article will be made available by the authors on request.

## References

[1] Gancz A.Y., Kistler A.L., Greninger A.L., Farnoushi Y., Mechani S., Perl S., Berkowitz A., Perez N., Clubb S., DeRisi J.L. (2009). Experimental induction of proventricular dilatation disease in cockatiels (*Nymphicus hollandicus*) inoculated with brain homogenates containing avian bornavirus 4. Virol. J..

[2] Hoppes S., Gray P.L., Payne S., Shivaprasad H.L., Tizard I. (2010). The isolation, pathogenesis, diagnosis, transmission, and control of avian bornavirus and proventricular dilatation disease. Vet. Clin. North. Am. Exot. Anim. Pract..

[3] Hoppes S.M., Tizard I., Shivaprasad H.L. (2013). Avian bornavirus and proventricular dilatation disease: Diagnostics, pathology, prevalence, and control. Vet. Clin. North. Am. Exot. Anim. Pract..

[4] Kistler A.L., Gancz A., Clubb S., Skewes-Cox P., Fischer K., Sorber K., Chiu C.Y., Lublin A., Mechani S., Farnoushi Y. (2008). Recovery of divergent avian bornaviruses from cases of proventricular dilatation disease: Identification of a candidate etiologic agent. Virol. J..

[5] Ouyang N., Storts R., Tian Y., Wigle W., Villanueva I., Mirhosseini N., Payne S., Gray P., Tizard I. (2009). Histopathology and the detection of avian bornavirus in the nervous system of birds diagnosed with proventricular dilatation disease. Avian Pathol..

[6] Piepenbring A.K., Enderlein D., Herzog S., Kaleta E.F., Heffels-Redmann U., Ressmeyer S., Herden C., Lierz M. (2012). Pathogenesis of avian bornavirus in experimentally infected cockatiels. Emerg. Infect. Dis..

[7] Leal de Araujo J., Rech R.R., Heatley J.J., Guo J., Giaretta P.R., Tizard I., Rodrigues-Hoffmann A. (2017). From nerves to brain to gastrointestinal tract: A time-based study of parrot bornavirus 2 (PaBV-2) pathogenesis in cockatiels (*Nymphicus hollandicus*). PLoS ONE.

[8] Donatti R.V., Resende M., Ferreira F.C., Marques M.V., Ecco R., Shivaprasad H.L., de Resende J.S., Martins N.R. (2014). Fatal proventricular dilatation disease in captive native psittacines in Brazil. Avian Dis..

[9] Keller D.L., Honkavuori K.S., Briese T., Lipkin W.I., Muthuswamy A., Steinberg H., Sladky K.K. (2010). Proventricular dilatation disease associated with avian bornavirus in a scarlet macaw (Ara macao). J. Vet. Diagn. Investig..

[10] Mirhosseini N., Gray P.L., Hoppes S., Tizard I., Shivaprasad H.L., Payne S. (2011). Proventricular dilatation disease in cockatiels (*Nymphicus hollandicus*) after infection with a genotype 2 avian bornavirus. J. Avian Med. Surg..

[11] Nedorost N., Maderner C.A., Kolodziejek J., Lussy H., Nowotny N., Weissenböck H. (2012). Identification of mixed infections with different genotypes of avian bornaviruses in psittacine birds with proventricular dilatation disease. Avian Dis..

[12] Ogawa H., Sanada Y., Sanada N., Kudo M., Tuchiya K., Kodama T., Uetsuka K. (2011). Proventricular dilatation disease associated with avian bornavirus infection in a Citron-crested Cockatoo that was born and hand-reared in Japan. J. Vet. Med. Sci..

[13] Rautenschlein S., Yeh H.Y., Sharma J.M. (2002). The role of T cells in protection by an inactivated infectious bursal disease virus vaccine. Vet. Immunol. Immunopathol..

[14] Rubbenstroth D., Rinder M., Stein M., Höper D., Kaspers B., Brosinski K., Horie M., Schmidt V., Legler M., Korbel R. (2013). Avian bornaviruses are widely distributed in canary birds (*Serinus canaria* f. domestica). Vet. Microbiol..

[15] Vaden S.L., Cullen J.M., Riviere J.E. (1995). Pharmacokinetics of cyclosporine in woodchucks and Pekin ducks. J. Vet. Pharmacol. Ther..

[16] Heffels-Redmann U., Enderlein D., Herzog S., Piepenbring A., Bürkle M., Neumann D., Herden C., Lierz M. (2012). Follow-up investigations on different courses of natural avian bornavirus infections in psittacines. Avian Dis..

[17] Kerski A., de Kloet A.H., de Kloet S.R. (2012). Vertical transmission of avian bornavirus in psittaciformes: Avian bornavirus RNA and anti-avian bornavirus antibodies in eggs, embryos, and hatchlings obtained from infected sun conures (*Aratinga solstitialis*). Avian Dis..

[18] Delnatte P., Ojkic D., Delay J., Campbell D., Crawshaw G., Smith D.A. (2013). Pathology and diagnosis of avian bornavirus infection in wild Canada geese (Branta canadensis), trumpeter swans (Cygnus buccinator) and mute swans (Cygnus olor) in Canada: A retrospective study. Avian Pathol..

[19] Payne S., Shivaprasad H.L., Mirhosseini N., Gray P., Hoppes S., Weissenbock H., Tizard I. (2011). Unusual and severe lesions of proventricular dilatation disease in cockatiels (*Nymphicus hollandicus*) acting as healthy carriers of avian bornavirus (PaBV) and subsequently infected with a virulent strain of PaBV. Avian Pathol..

[20] Schrank C.S., Cook M.E., Hansen W.R. (1990). Immune response of mallard ducks treated with immunosuppressive agents: Antibody response to erythrocytes and in vivo response to phytohemagglutinin-P. J. Wildl. Dis..

[21] Last R.D., Weissenböck H., Nedorost N., Shivaprasad H.L. (2012). Avian bornavirus genotype 4 recovered from naturally infected psittacine birds with proventricular dilatation disease in South Africa. J. S. Afr. Vet. Assoc..

[22] Hoppes S., Tizard I., Shivaprasad H.L., Heatley J.J. Treatment of Avian Bornavirus-infected Cockatiels (*Nymphicus hollandicus*) with Oral Meloxicam and Cyclosporine. Proceedings of the 33rd Annual Conference of the Association of Avian Veterinarians 2012.

[23] Dennison S.E., Paul-Murphy J.R., Adams W.M. (2008). Radiographic determination of proventricular diameter in psittacine birds. J. Am. Vet. Med. Assoc..

[24] Hoppes S., Heatley J.J., Guo J., Turner D., Shivaprasad H.L., Tizard I. (2013). Meloxicam treatment in cockatiels (*Nymphicus hollandicus*) infected with avian bornavirus. J. Exot. Pet Med..

[25] Planz O., Bilzer T., Stitz L. (1995). Immunopathogenic role of T-cell subsets in Borna disease virus-induced progressive encephalitis. J. Virol..

[26] Bilzer T., Stitz L. (1993). Brain cell lesions in Borna disease are mediated by T cells. Arch. Virol. Suppl..

[27] Staeheli P., Rinder M., Kaspers B. (2010). Avian bornavirus associated with fatal disease in psittacine birds. J. Virol..

[28] Stitz L., Bilzer T., Richt J.A., Rott R. (1993). Pathogenesis of Borna disease. Arch. Virol. Suppl..

[29] Fite K.V., Pardue S., Bengston L., Hayden D., Smyth J.R. (1986). Effects of cyclosporine in spontaneous, posterior uveitis. Curr. Eye Res..

[30] Fitzgerald S.D., Reed W.M., Furukawa A.M., Zimels E., Fung L. (1995). Effect of T-lymphocyte depletion on the pathogenesis of marble spleen disease virus infection in ring-necked pheasants. Avian Dis..

[31] Ganapathy K., Bradbury J.M. (2003). Effects of cyclosporin A on the immune responses and pathogenesis of a virulent strain of Mycoplasma gallisepticum in chickens. Avian Pathol..

[32] Hill J.E., Rowland G.N., Latimer K.S., Brown J. (1989). Effects of cyclosporine A on reovirus-infected broilers. Avian Dis..

[33] Isobe T., Shimizu S., Yoshihara S., Yokomizo Y. (2000). Cyclosporin A, but not bursectomy, abolishes the protective immunity of chickens against Leucocytozoon caulleryi. Dev. Comp. Immunol..

[34] Khehra R.S., Jones R.C. (1999). Investigation into avian pneumovirus persistence in poults and chicks using cyclosporin A immunosuppression. Res. Vet. Sci..

[35] Kim Y., Brown T.P., Pantin-Jackwood M.J. (2003). Effects of cyclosporin A treatment on the pathogenesis of avian leukosis virus subgroup J infection in broiler chickens with Marek’s disease virus exposure. J. Vet. Sci..

[36] Kogut M.H., Eirmann L. (1991). The effect of cyclosporin A on the development of Eimeria in non-specific hosts. Int. J. Parasitol..

[37] Loa C.C., Lin T.L., Wu C.C., Bryan T., Hooper T., Schrader D. (2002). The effect of immunosuppression on protective immunity of turkey poults against infection with turkey coronavirus. Comp. Immunol. Microbiol. Infect. Dis..

[38] Pantin-Jackwood M.J., Brown T.P., Kim Y., Huff G.R. (2004). Proventriculitis in broiler chickens: Effects of immunosuppression. Avian Dis..

[39] Plumb DCCyclosporine In: Plumb D.C., Davidson G. (2011). Plumb’s Veterinary Drug Handbook.

[40] Pons H.A., Adams S., Stadecker M.J. (1988). Schistosoma mansoni: The basis for the antischistosomal effect of cyclosporine A. Exp. Parasitol..

[41] Rubbenstroth D., Schmidt V., Rinder M., Legler M., Corman V.M., Staeheli P. (2014). Discovery of a new avian bornavirus genotype in estrildid finches (Estrildidae) in Germany. Vet. Microbiol..

[42] Russell P.H., Dwivedi P.N., Davison T.F. (1997). The effects of cyclosporin A and cyclophosphamide on the populations of B and T cells and virus in the Harderian gland of chickens vaccinated with the Hitchner B1 strain of Newcastle disease virus. Vet. Immunol. Immunopathol..

[43] Stitz L., Soeder D., Deschl U., Frese K., Rott R. (1989). Inhibition of immune-mediated meningoencephalitis in persistently Borna disease virus-infected rats by cyclosporine A. J. Immunol..

[44] Suresh M., Sharma J.M. (1995). Hemorrhagic enteritis virus induced changes in the lymphocyte subpopulations in turkeys and the effect of experimental immunodeficiency on viral pathogenesis. Vet. Immunol. Immunopathol..

[45] Tully T.N., Mitchell M., Tully T.N. (2009). Birds: Physical examination and restraint. Manual of Exotic Pet Practice.

[46] Kramer M.R., Marshall S.E., Denning D.W., Keogh A.M., Tucker R.M., Galgiani J.N., Lewiston N.J., Stevens D.A., Theodore J. (2015). Cyclosporine and itraconazole interaction in heart and lung transplant recipients. Ann. Intern. Med..

[47] Weissenböck H., Sekulin K., Bakonyi T., Högler S., Nowotny N. (2009). Novel avian bornavirus in a nonpsittacine species (Canary; *Serinus canaria*) with enteric ganglioneuritis and encephalitis. J. Virol..

[48] Li Y., Sun S., Guo Q., Ma L., Shi C., Su L., Li H. (2008). In vitro interaction between azoles and cyclosporin A against clinical isolates of Candida albicans determined by the chequerboard method and time–kill curves. J. Antimicrob. Chemother..

[49] Antonissen G., Devreese M., De Baere S., Martel A., Goessens T., Haesendonck R., De Backer P., Croubels S. Pharmacokinetics of the selective COX-2 inhibitors celecoxib and mavacoxib in cockatiels. Proceedings of the 13th International congress of the European Association for Veterinary Pharmacology and Toxicology 2015.

[50] Escandon P. (2015). Nonsteroidal Anti-Inflammatory Drug Treatment of Cockatiels (Nymphicus hollandicus) Experimentally Inoculated with Parrot Bornavirus 2. Doctoral Dissertation.

[51] Hedau M., Bhandarkar A.G. (2016). Pathology of Meloxicam Toxicity in Cockerel Birds. Indian. Vet. J..

[52] Carlos C.P., Sonehara N.M., Oliani S.M., Burdmann E.A. (2014). Predictive usefulness of urinary biomarkers for the identification of cyclosporine A-induced nephrotoxicity in a rat model. PLoS ONE.

